# Genetic Variation of the Endangered Neotropical Catfish *Steindachneridion scriptum* (Siluriformes: Pimelodidae)

**DOI:** 10.3389/fgene.2018.00048

**Published:** 2018-02-19

**Authors:** Rômulo V. Paixão, Josiane Ribolli, Evoy Zaniboni-Filho

**Affiliations:** ^1^Laboratório de Biologia e Cultivo de Peixes de Água Doce, Departamento de Aquicultura, Universidade Federal de Santa Catarina, Florianópolis, Brazil; ^2^Programa de Pós-Graduação em Aquicultura, Centro de Ciências Agrárias, Universidade Federal de Santa Catarina, Florianópolis, Brazil

**Keywords:** control region mitochondrial DNA, conservation of natural resources, DNA barcode, endangered species, freshwater fishes

## Abstract

*Steindachneridion scriptum* is an important species as a resource for fisheries and aquaculture; it is currently threatened and has a reduced occurrence in South America. The damming of rivers, overfishing, and contamination of freshwater environments are the main impacts on the maintenance of this species. We accessed the genetic diversity and structure of *S. scriptum* using the DNA barcode and control region (D-loop) sequences of 43 individuals from the Upper Uruguay River Basin (UUR) and 10 sequences from the Upper Paraná River Basin (UPR), which were obtained from GenBank. *S. scriptum* from the UUR and the UPR were assigned in two distinct molecular operational taxonomic units (MOTUs) with higher inter-specific K2P distance than the optimum threshold (OT = 0.0079). The COI Intra-MOTU distances of *S. scriptum* specimens from the UUR ranged from 0.0000 to 0.0100. The control region indicated a high number of haplotypes and low nucleotide diversity, compatible with a new population in recent expansion process. Genetic structure was observed, with high differentiation between UUR and UPR basins, identified by BAPS, haplotype network, AMOVA (*F*_ST_ = 0.78, *p* < 0.05) and Mantel test. *S. scriptum* from the UUR showed a slight differentiation (*F*_ST_ = 0.068, *p* < 0.05), but not isolation-by-distance. Negative values of Tajima’s *D* and Fu’s *F*s suggest recent demographic oscillations. The Bayesian skyline plot analysis indicated possible population expansion from beginning 2,500 years ago and a recent reduction in the population size. Low nucleotide diversity, spatial population structure, and the reduction of effective population size should be considered for the planning of strategies aimed at the conservation and rehabilitation of this important fisheries resource.

## Introduction

Freshwater ecosystems are among the most endangered ecosystems (Dudgeon et al., 2006). Habitat degradation, hydrologic alterations, habitat fragmentation, sediment deposition, and overfishing are the principal causes of declines and extinctions of freshwater fishes (Dudgeon et al., 2006; Agostinho et al., 2008; Helfman, 2008; Hoeinghaus et al., 2009). In addition, species with geographically restricted distribution are more susceptible to erosion of the genetic diversity due to habitat fragmentation (Vrijenhoek et al., 1985). Fishery resources are an integral part of most societies and make important contributions to economic and social health and well-being in many countries and areas (FAO, 2002). The understanding of the genetic diversity and structure of wild populations of fish species are important to the regulation of fisheries and conservation management strategies (Carvalho and Hauser, 1994; Iervolino et al., 2010).

*Steindachneridion scriptum* (Miranda Ribeiro, 1918) is a large catfish belonging to the family Pimelodidae. This potamodromous fish species presents restricted distribution in the Upper Uruguay River (UUR) and Upper Paraná River (UPR) basins (Marques et al., 2002). *S. scriptum* is an important fishing resource to the riverine fishermen (Schork et al., 2013); nonetheless, it was recently classified as an endangered species according to the Chico Mendes Institute for Biodiversity Conservation (ICMBIO, 2014). Human activities (e.g., damming of rivers, illegal fishing, and industrial waste) are the main threats to *S. scriptum* in the UUR (Fontana et al., 2003) and are the principle reason for population reduction in this basin (Beux and Zaniboni-Filho, 2008).

The correct management of fish stocks depends on the precise identification of the target species, which may present very similar morphological characteristics to other species. The latest revision of the genera *Steindachneridion* recognizes six valid species (Garavello, 2005). Using museum specimen, Garavello (2005) suggests the presence of *S. punctatum* in the UUR and UPR with few characteristics that differ from *S. scriptum*. Despite the description of the two species of the genus *Steindachneridion*, ichthyofauna studies never report the presence of *S. punctatum* in the UUR (Schork et al., 2012, 2013). In addition to the correct ichthyofauna and management of fish stocks, uncertainties of taxonomic identification may be a problem for stock management (e.g., formation of *in vivo* and *in vitro* banks, restocking programs) and adequate fisheries control.

Taxonomic uncertainties are common in fishes (Pereira et al., 2013) and can be investigated using DNA barcode methodology, which permits the unambiguous identification of the majority of fish species (Ward, 2009). Particularly for endangered species, prior knowledge of the distribution of genetic variability within and among natural populations as well as the implementation of an efficient management plan based on genetic features are important measures for its maintenance and recovery (Cross, 2000). The distribution of the genetic variation within and between populations can be assessed using the mitochondrial control region (Sivasundar et al., 2001; Iervolino et al., 2010; Ochoa et al., 2015). Given these findings, we tested the null hypothesis that *S. scriptum* from UUR represent a single molecular operational taxonomic unit (MOTU). Posteriorly, we investigate the genetic diversity and population structure of *S. scriptum* from the UUR and UPR basins using the mitochondrial control region.

## Materials and Methods

### Study Area and Sampling

**FIGURE 1:**
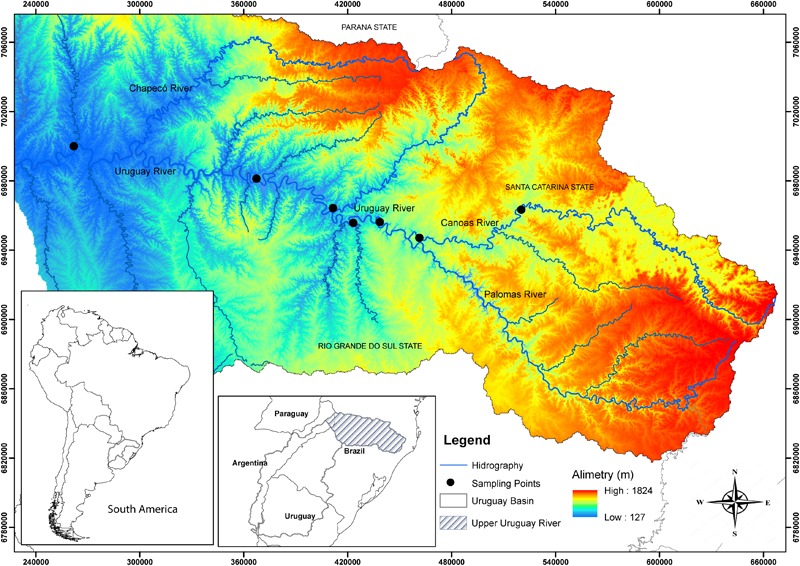
Map showing the locations of sampling sites of the *Steindachneridion scriptum* in the Upper Uruguay River Basin. Black dots indicate sample points.

The Uruguay River (Uru) originates in Brazilian territory (in the Serra Geral Mountains) in Southern Brazil, together with the Paraná and Paraguay Rivers form the La Plata Basin (Zaniboni-Filho and Schulz, 2003). Samples of *S. scriptum* were collected by scientific fishing and local fishermen between 2006 and 2015, with authorization of the Brazilian Institute of the Environment and Renewable Natural Resources (IBAMA; protocol number: 02026.005762/2004-71). A total of 19 individuals from the Uru River and 24 from the Canoas River (Can), UUR basin, were sampled (**Figure 1**). Tissues were preserved in 95% ethanol until extraction. This research was conducted under Animal Care Protocol PP00788 of the Federal University of Santa Catarina (UFSC).

### DNA Extraction and Amplification of the Mitochondrial Fragments

Total DNA was obtained with fin clips following a salt extraction method (Aljanabi and Martinez, 1997). For the DNA barcode analyses, a fragment of 652 bp of Cytochrome Oxidase subunit I (COI) was amplified through polymerase chain reaction (PCR), using primers FishF1/FishR1 (Ward et al., 2005) and following Bellafronte et al. (2013). The partial amplification of the mitochondrial control region (D-loop) was performed using primers FTTP-L and DLR1-H according to Huergo et al. (2011). The PCR products were checked for amplification using gel electrophoresis with 1% agarose gels purified using PEG 20% (Lis, 1980). Sequencing reactions were performed using BigDye TM Terminator v 3.1 (Applied Biosystems), and the PCR products were sequenced for both strands in ABI 3500XL (Applied Biosystems).

### Data Analysis

DNA sequences from each individual of both genes were edited using Geneious 5.4.4 (Wu and Drummond, 2011) to generate a consensus sequence. For the DNA barcode analysis, we combined the COI reference sequences of *S. scriptum* (access FUPR686-09, PDCAP027-14, and PDCAP028-14) from the UPR basin, *S. parahybae* (access FPSR293-10–FPSR297-10) from the Paraíba do Sul Basin and one specimen of *Pseudoplatystoma corruscans* and one specimen of *Zungaro jahu* to root our phylogenetic analyses. All sequences available in Barcode of Life Data System (BOLD). Intra- and inter-specific genetic distances based on the Kimura 2-parameter (K2P) evolution model were calculated using Mega 6 (Tamura et al., 2013).

We used the phylogenetic General Mixed Yule Coalescent (GMYC) approach based on single-locus data that is a relative robust tool for species delimitation (Pons et al., 2006; Fujisawa and Barraclough, 2013). The ultrametric tree was generated in BEAST v.2.2.1 (Bouckaert et al., 2014), with the substitution model calculated in the JModelTest 2.1.4 (HKY+G; Darriba et al., 2012), using relaxed molecular clock with a lognormal distribution and birth–death model. Three independent runs were carried out with 20 million generations each. Posteriorly, the runs were combined using the LogCombiner v.1.8.3 (Drummond et al., 2012), with a burn-in of 25. Data mixing and effective sample size (ESS) were verified in Tracer v1.5. GMYC was carried out in Species Limits by Threshold Statistics (SPLITs; Monaghan et al., 2009) with RStudio^1^, using the unique threshold method to detect the transition point between intra- and inter-specific relationships.

In addition to the standard threshold adopted to Neotropical fishes (Pereira et al., 2013), we calculated an optimum threshold (OT; Collins et al., 2012) directly from all dataset, using the *local minima* function in the R package SPIDER (SPecies IDentity and Evolution in R; Brown et al., 2012). The OT value was used to define the MOTUs using the software jMOTU (Jones et al., 2011). The graphical representation of the MOTUs was performed by a neighbor-joining analysis (NJ) using the K2P model with Mega 6.6 (Tamura et al., 2013). The support of the clades was tested by the bootstrap method with 10,000 pseudo-replicates.

DNA D-loop sequences were combined with 16 *S. scriptum* sequences downloaded from GenBank, of specimens collected between 1995 and 2002 that corresponded to the Uru River (access EU930029.1–EU930038.1) and specimens from Tibaji River (UPR) (access EU930039.1–EU930044.1). The overall genetic diversity was estimated using the following DnaSP software (Rozas et al., 2003) parameters: nucleotide diversity (π) (Nei, 1987), haplotype diversity (Hd) (Nei and Tajima, 1981), and number of polymorphic sites (*S*). Genetic diversity within and between sample sites was hierarchically tested by Analysis of Molecular Variance (AMOVA) (Excoffier et al., 1992) with 10,000 permutations to test the pairwise population comparison (*F*_ST_) using Arlequin 3.1 (Excoffier and Lischer, 2010). Spatial genetic structure was inferred using Bayesian Analysis of Population Structure 6.0 (BAPS) software (Corander et al., 2013). First, BAPS was run with 10 replicates for every level of *k* (1–6) without origin information (“clustering of individuals”) and subsequently using “clustering of groups of individuals.” We used Mantel tests as implemented in the Alleles In Space (AIS) 1.0 (Miller, 2005) to test for a correlation of geographic stream distance and genetic distance (isolation-by-distance; IBD) expressed as *F*_ST_, with 10,000 permutations to assess significance. Tajima’s (1989) *D*, Fu’s (1997) *F*s, and mismatch distributions were estimated with *DnaSP* (Rozas et al., 2003). A median-joining haplotype network was generated through PopART (Bandelt et al., 1999). Demographic history was investigated using Bayesian Skyline Plot (BSP) in BEAST 2.1.3 (Drummond et al., 2012) with the evolutionary model obtained in the Jmodeltest program. The graphic was generated in Tracer 1.66 (Rambaut et al., 2014).

The COI sequences were deposited in BOLD systems (accession UUR001-17–UUR052-17), and DNA D-loop sequences were uploaded in GenBank (accession MF045370–MF045412). The voucher corresponding to *S. scriptum* from the UUR basin was deposited in the Zoology Museum of the Universidade Estadual de Londrina MZUEL 15569 (Type locality: Itaqui, RS – Uru Basin).

## Results

### Mitochondrial DNA Barcoding

The consensus alignment of 43 COI sequences were obtained from samples identified morphologically as *S. scriptum*, resulting in a total length of 611 bp, with 7 polymorphic sites and 6 haplotypes defined. The inter-specific nucleotide frequencies were 26.02% of Cytosine, 28.97% of Thymine, 27.19% of Adenine, and 17.82% of Guanine. No stop codons, insertions, or deletions were observed in the COI sequences, indicating that they represent fragments of functional mitochondrial genes and not nuclear mitochondrial pseudo-genes (Numts). Considering *P. corruscans* and *Z. jahu* as the outgroup, the maximum likelihood for the GMYC model was significantly superior (*L* = 628.3242) to the likelihood of the null model (*L*o = 614.7156, *p* < 0.0001). The single-threshold GMYC model suggested the presence two clusters (confidence interval 2–13) of four ML entities (GMYC ‘species,’ named ‘MOTUs’ herein) with a confidence interval of 4–17 (*S. scriptum, S. parahybae*, and two outgroups).

The optimal threshold calculated for all *S. scriptum* sequences used in this study was OT = 0.0079 (0.79%) of divergence. From the set of the genus *Steindachneridion* sequences available on BOLD, three MOTUs were identified using the OT value and software jMOTU: *S. scriptum* from the UUR, *S. scriptum* from the UPR, and *S. parahybae*. The COI inter-MOTU between *S. scriptum* from the UUR and *S. scriptum* from the UPR showed values larger than the OT (mean 0.012, minimum 0.010), while intra-MOTU values for fishes from both basins were lower than the average OT (UUR = 0.000 to 0.010; UPR = 0.000) (**Table 1**). The minimum inter-specific distances between *S. scriptum* MOTUs (UUR and UPR) and *S. parahybae* were 0.100 (10%) and 0.090 (9%), respectively.

**Table 1:** Intra-MOTU distances (in bold) and inter-MOTU genetic distances using the COI gene and K2p model.

	1	2	3
1 *S. scriptum* UUR	**0.001 (0.000–0.010)**		
2 *S. scriptum* UPR	0.012 (0.010–0.017)	**0.000 (0.000)**	
3 *S. parahybae*	0.102 (0.100–0.106)	0.090 (0.090)	**0.003 (0.000–0.003)**

*Distance range in parentheses. UUR = Upper Uruguay River; UPR = Upper Paraná River.*

**FIGURE 2:**
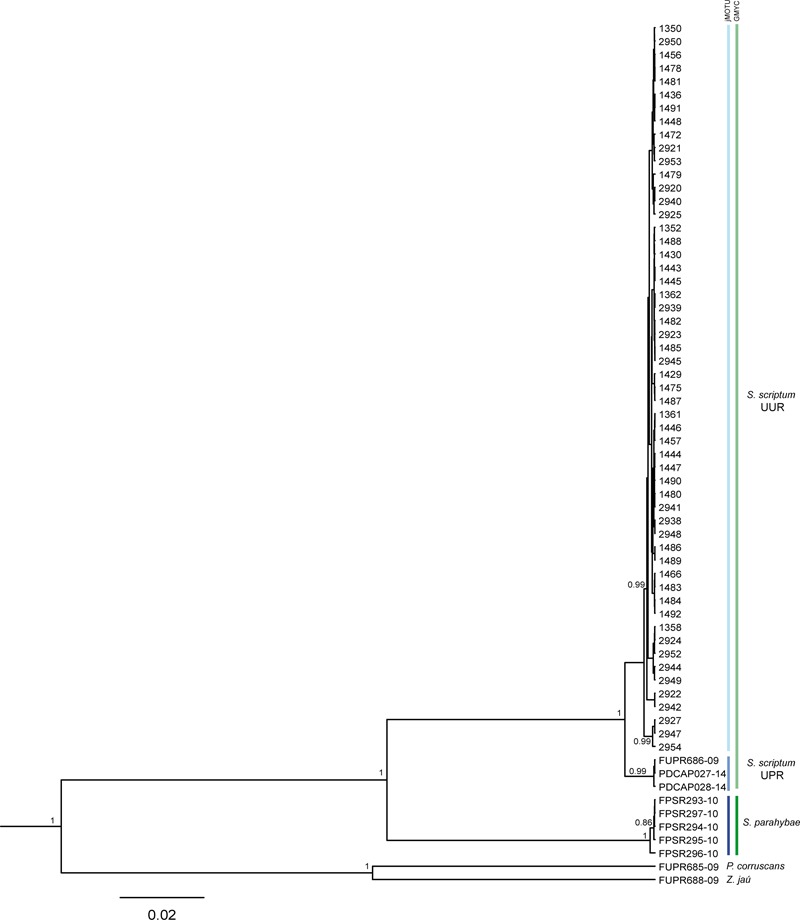
Phylogenetic tree of the genus *Steindachneridion* based on the mitochondrial gene COI inferred with BEAST. Values before nodes are Bayesian posterior probabilities. Codes FUPR, PDCAP, and FPSR were downloaded from the BOLD system. *Pseudoplatystoma corruscans* and *Z. jahu* are outgroups. Vertical lines in Blue = jMOTU MOTUs, and vertical lines in Green indicate GMYC MOTUs.

The NJ-K2P (Supplementary Figure SI 1) and Bayesian Inference topologies (**Figure 2**) were clustered in two principal clusters, corresponding to *S. scriptum* and *S. parahybae* species. The *S. scriptum* clade appeared divided into two well-supported sub-clades formed by UUR and UPR individuals, in both NJ and BI phylogenetic trees. Based on these results, all specimens from Uru were composed of a single MOTU, named here as *S. scriptum*.

### Mitochondrial DNA D-Loop

**FIGURE 3:**
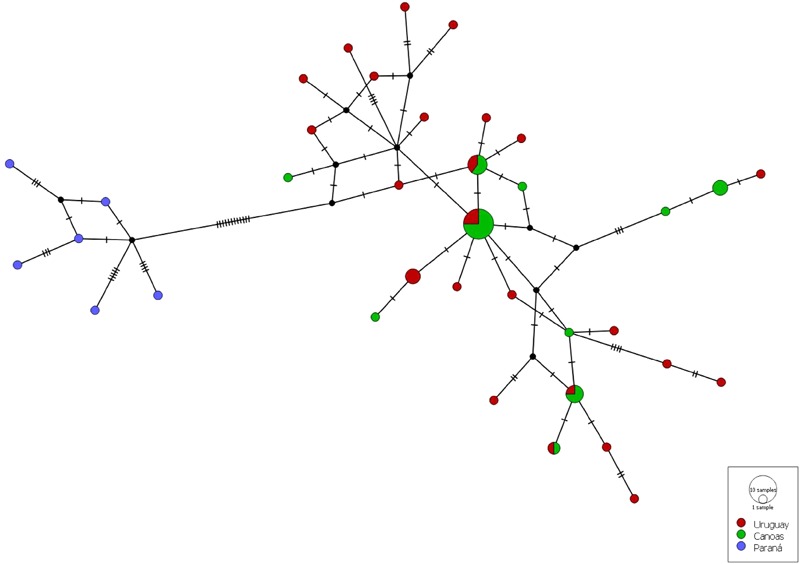
Median-joining network of *Steindachneridion scriptum*, based on haplotypes of mtDNA control region. The colors indicate locality according to the legend, size of the circles illustrates the number of identical haplotypes, and small black circles, hypothetical ancestors or unsampled haplotypes. Hatch marks represent the number of mutations by which haplotypes differ.

The final alignment size of the 59 D-loop consensus sequences of *S. scriptum* specimens from the UUR and UPR (43 newly sequenced, 16 downloaded from GenBank) was 865 bp. A total of 56 variable sites were found in the region defining a total of 36 haplotypes. Overall, 30 haplotypes identified in the analyses were unique and exclusive. Of these, 20 were from the Uru, 4 from Can, and 6 from UPR basin (Tibagi River). The most common haplotype was H8, which was recorded 12 times and was shared by samples from the Uru and Can rivers, and haplotypes H4 and H27, both with three records, were exclusive to the Uru and Can rivers, respectively. The haplotype network (**Figure 3**) revealed a high degree of similarity between the specimens from the Uru and Can rivers, even though with slight differences in the haplotype frequencies and exclusive haplotypes from each river. These specimens were also differentiated from the samples of UPR by 12 mutations.

The average nucleotide frequencies were 34.38% of Adenine, 33.17% of Thymine, 12.19% of Guanine, and 20.26% of Cytosine. Genetic variability, expressed as Hd) and nucleotide diversity (π), was higher in *S. scriptum* from the Uru (Hd = 0.959/π = 0.007) in comparison with the Can River (Hd = 0.837/π = 0.004) (**Table 2**). In addition, the samples from UPR have high diversity indices, in comparison with samples from UUR (*N* = 6; *N*h = 6; Hd = 1.0000, π = 0.00698; *D* = -0.88901, *p* = 0.24002; *F*s = -1.81313, *p* = 0.07768).

**Table 2:** Genetic diversity of S*teindachneridion scriptum* from the Uruguay and Canoas rivers (Upper Uruguay River Basin) estimated using D-loop control region.

Sampling Site	*N*	*N*h	Hd	π	*D*	*F*s
Canoas River	24	12	0.837	0.004	-0.049	-2.223
Uruguay River	29	23	0.959	0.007	-2.016^∗^	-16.346^∗^
Total	53	32	0.932	0.005	-1.907^∗^	-19.246^∗∗^

*N = number of sequenced individuals; *N*h = number of haplotypes; Hd = haplotype diversity; π = nucleotide diversity; *D* = Tajima’s *D*-test; *F*s = Fu’s *F*s-test. Significant values to *D*-test and *F*s-test: ^∗^*p* < 0.05, ^∗∗^*p* < 0.01.*

**FIGURE 4:**
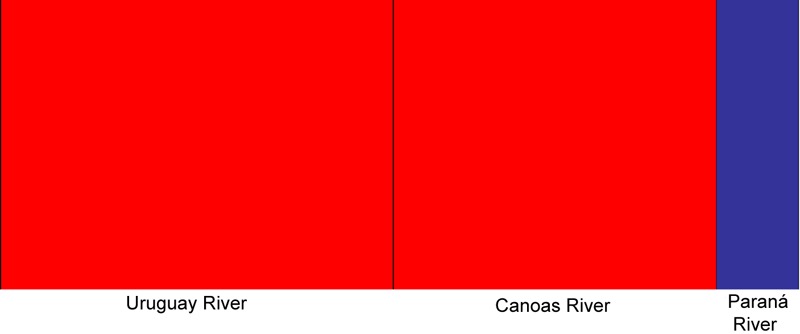
Estimate of the probable groups of populations produced by the BAPS between *Steindachneridion scriptum* from Upper Uruguay and Upper Parana Basins, assigned to two clusters (*K* = 2).

The patterns of genetic variability found within and between populations in the AMOVA were based on the two principal clusters: Uruguay vs. Paraná Basins and Uru vs. Can rivers (**Table 3**). When the populations were considered as two basin groups, the AMOVA among groups was 78.17% and *F*_ST_ value was highly significant (*F*_ST_ = 0.781; *p* = 0.000). Genetic divergence between individuals from the Uruguay Basin (Uru vs. Can rivers) was low but significant (*F*_ST_ = 0.0682, *p* = 0.00475). The population groupings generated by the BAPS, without origin information of the samples, revealed the existence of three clusters (*K* = 3, Supplementary Figure SI 2), with slight differentiation between Can and Uru individuals. On the other hand, analysis with the individuals identified by sample group indicated two clusters (*K* = 2, **Figure 4**), corresponding to Uruguay and Paraná Basins. The IBD analysis showed a significant positive correlation (*r*^2^ = 0.85, *p* < 0.001) between the geographical distance and corresponding *F*_ST_ for *S. scriptum* from UUR and UPR (Supplementary Figure SI 3). On the other hand, *F*_ST_ values plotted over distance no reveal patter of isolation by distance for S. scriptum from UUR (*r* = -0.052, *p* = 0.682).

**Table 3:** Hierarchical AMOVA analysis and *F*_ST_ values for S*teindachneridion scriptum* according to their geographical location, estimated using D-loop control region.

Type of variation	Component of variation	% of variation	*F*_ST_ (*p*)
**UUR × UPR**			
Between Basins	9.22585	78.17	0.78167^∗^(0.0000)
Within Basins	2.57696	21.83	
**Upper Uruguay Basin**			
Between populations	0.17916	6.83	0.06825^∗^ (0.0047)
Within populations	2.44580	93.17	

*UUR = Upper Uruguay River; UPR = Upper Parana River. ^∗^Significant value: *p*-value (*F*_*ST*_) = < 0.05.*

Tajima’s (1989) *D*-neutrality tests, applied to detect evidence of strong selective pressures, and Fu’s (1997) *F*s-tests, used specifically to detect population expansion, revealed significant negative values for all individuals from the UUR (*D* = -1.907, *p* < 0.05; *F*s = -19.246, *p* < 0.01; **Table 3**). Non-significant negative values were estimated for *D* and *F*s indexes for specimens from the Can River and UPR, whereas fish sampled in the Uru showed significant negative values estimated for *D* and *F*s indexes. The BSP analysis (Supplementary Figure SI 4), used to explore previous demographic signals of *S. scriptum*, indicated early demographic expansion approximately 2,500 years ago as well as a fairly recent population reduction.

## Discussion

The DNA barcode confirmed the identification of all the individuals of *S. scriptum* from UUR as a single MOTU. The different methods, jMOTU and GMYC, were congruent in delimiting *S. scriptum* and *S. parahybae*. However, within *S. scriptum*, jMOTU methodology identified two distinct MOTUs between UUR basin and UPR basin, while GMYC only one, despite strongly supported clade. Inter-MOTU divergence between *S. scriptum* from the UUR and the UPR was higher than the OT and the mean intra-specific divergence found for freshwater fish (0.3%) (Ward et al., 2005; Lara et al., 2010; Pereira et al., 2013). The MOTUs do not necessarily represent species (Blaxter et al., 2005) but can indicate molecular entities (Casiraghi et al., 2010). The two MOTUs estimated between *S. scriptum* from the UUR and *S. scriptum* from the UPR could be explained by geographic isolation between watersheds that occurred during the Miocene epoch (between 5 and 24 million years ago) when the UUR and the UPR became isolated (Albert and Reis, 2011). Based on the results, *S. scriptum* from these two hydrographic systems are most likely in the process of incipient allopatric speciation since the genetic structuring in fish is in fact often evidenced and influenced principally by geological, ecological, and behavioral factors (Allan and Flecker, 1993). Congeners *S. scriptum* and *S. parahybae* species showed mean inter-MOTUs 10 times greater than the OT, indicating the existence of the barcode gap (Hebert et al., 2003, 2004) that allows us to assign an unknown *Steindachneridion* specimen to its species using a genetic distance criterion with an insignificant error rate.

Although they belong to the same watershed, individuals from the Can River and the Uru River showed a slight genetic differentiation probably due to the topography of the region and the interaction between the species’ biology and environmental characteristics (e.g., the Can River is located at a higher altitude with a lower water temperature than the Uru River). The haplotype network indicated a greater genetic similarity between the specimens from the Can and Uru, whereas is possible to observe differences in the haplotype frequencies, and exclusive haplotypes for each river. Recent studies with potamodromous fish species reported genetic structure in hydrographic systems without apparent physical barriers, resulting from behaviors related to IBD (Hardy and Vekemans, 1999; Primmer et al., 2006; Han et al., 2010), *homing* (Windle and Rose, 2005; Batista and Alves-Gomes, 2006; Neville et al., 2006), and isolation-by-time (IBT) (Hendry and Day, 2005; Braga-Silva and Galetti, 2016; Ribolli et al., 2017).

Nucleotide diversity of *S. scriptum* from the UUR was low in comparison with the values found in neotropical freshwater fish (π = 1.5%) (Batista and Alves-Gomes, 2006; Iervolino et al., 2010; Ashikaga et al., 2015). The high haplotype diversity and low nucleotide diversity seem quite compatible with a new population in recent expansion process, similar to what is shown in BSP analyses. This pattern may be a signature of such expansion that is long enough to examine a change in the haplotypes resulted from the mutation, but is not long enough to accumulate large differences between sequences (Avise, 2000).

Individuals from the Uru River were genetically more diverse than fishes from the Can River, indicating that the main channel of the Uru River allows the meeting of individuals of different areas (or tributaries), favoring the maintenance of the highest level of genetic diversity. Although it is a relevant fishing resource, useful molecular markers such as microsatellites, extensively employed in fish genetic studies, are still not developed for any *Steindachneridion* species, and the knowledge about genetic characteristics of the genus is incipient. RAPD markers indicated low genetic diversity for *S. scriptum* from the UUR, as reported by Ramella et al. (2006), as well as for *S. melanodermatum* from the Iguaçu River Basin (Matoso et al., 2011). Low genetic diversity may indicate recent or historic reduction of this diversity; however, some endangered populations may have a historic maintenance of small effective population sizes (Matocq and Villablanca, 2001). In this way, the low diversity detected in *S. scriptum* from the UUR can be attributed to the following: (1) population history of this species: a Bayesian skyline plot analysis revealed a subtle increase in effective population size over time and demographic swings in the recent past, with a notable increase in effective population size between 1,000 and 2,500 years ago and a subsequent reduction in the effective size of females; and (2) evolutionary history of the species: according to Garavello (2005) and Swarça et al. (2005), *S. scriptum* notably maintain conserved morphological and cytogenetic patterns.

In general, concerns and actions of conservation are more related to the perception of the disappearance of a given species than of genetic diversity reduction (Frankham, 2005). Therefore, given the low genetic diversity associated with the current scenario of fragmentation of the UUR basin and the population reduction of *S. scriptum* in some stretches of the Itá and Machadinho reservoirs (Schork et al., 2012, 2013), this study highlights the necessity of mitigation measures and more intense monitoring of illegal fishing to avoid the collapse of this important fishing resource. In addition, our results were congruent, identifying great differentiation between individuals from the UPR and UUR Basins. Further studies with a larger number of samples morphological analyze to better define the taxonomic status of endangered *S. scriptum*.

## Author Contributions

RP made substantial contributions to the design of the work and acquisition, analysis, and interpretation of data for the work, and drafted it until the approval for publication of the content. JR made substantial contributions to the conception and design of the work, analysis and interpretation of the data, and drafted and revised it critically for important intellectual content. EZ-F made substantial contributions to the conception of the work, revised it critically for important intellectual content, and provided approval for publication of the content. The authors agreed to be accountable for all aspects of the work in ensuring that questions related to the accuracy or integrity of any part of the work are appropriately investigated and resolved.

## Conflict of Interest Statement

The authors declare that the research was conducted in the absence of any commercial or financial relationships that could be construed as a potential conflict of interest.

## Footnotes

1 http://r-forge.r-project.org/projects/splits
