# Supplementary material for: Genetic Variation of the Endangered Neotropical Catfish Steindachneridion scriptum (Siluriformes: Pimelodidae)
Source: Front Genet. 2018 Feb 19;9:48. doi: 10.3389/fgene.2018.00048 (PMC5827538; doi:10.3389/fgene.2018.00048)
Supplement: Supplementary file 1 [file Image_1.pdf]

Supplementary Material

Genetic variation of the endangered Neotropical catfish *Steindachneridion scriptum* (Siluriformes: Pimelodidae)

Rômulo Veiga Paixão*, Josiane Ribolli, Evoy Zaniboni Filho

*** Correspondence:** Corresponding Author: romulo.veiga.paixao@gmail.com

1 Supplementary Information - SI

SI 1. Neighbor-joining phylogenetic tree based on COI sequences of Steindachneridion scriptum from Upper Uruguay and Upper Paraná Basins, S. parahybae from Paraíba do Sul Basin and P. corruscans and Z. jahu as outgroup.

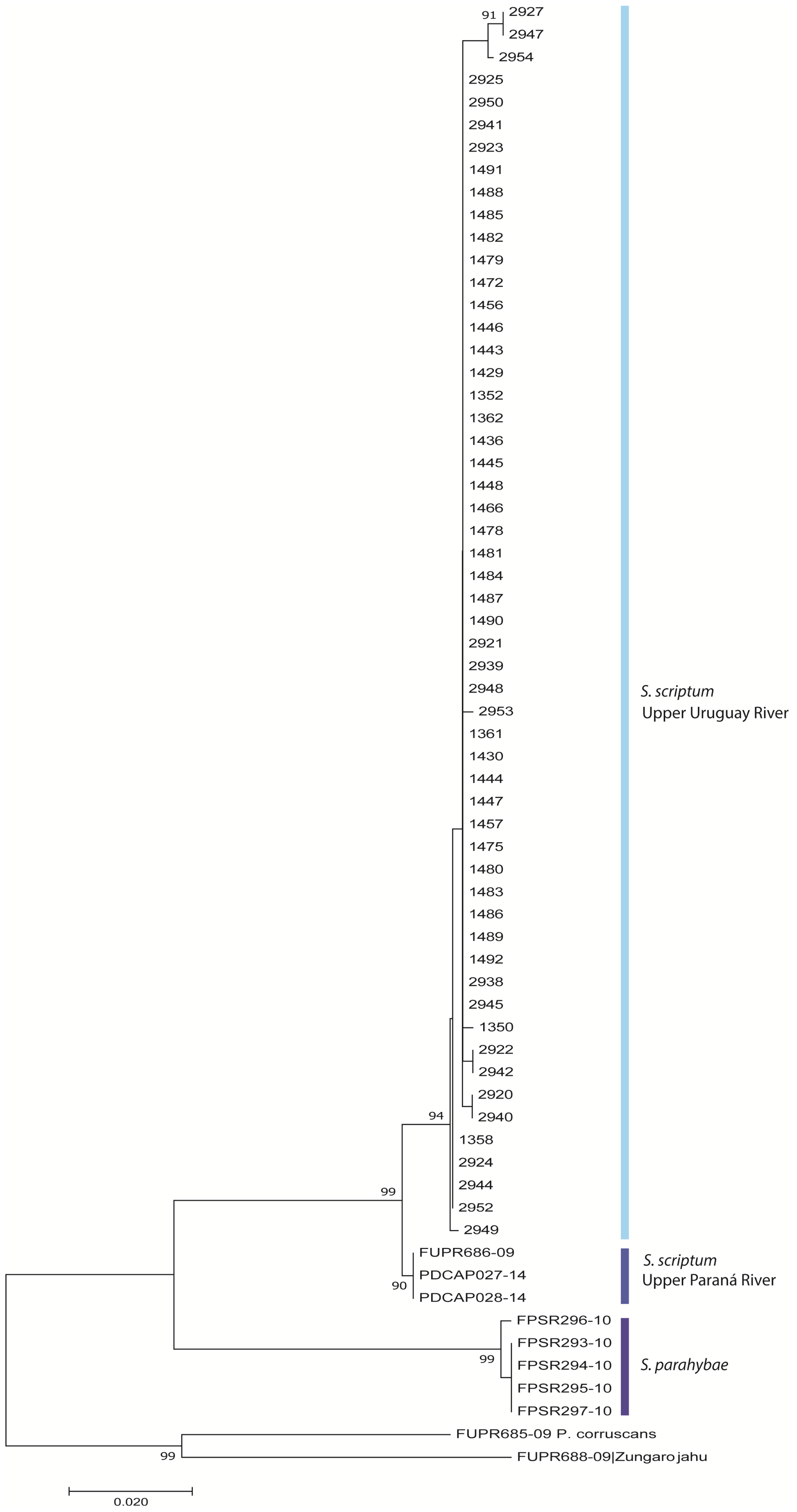
91
2927
2947
2954
2925
2950
2941
2923
1491
1488
1485
1482
1479
1472
1456
1446
1443
1429
1352
1362
1436
1445
1448
1466
1478
1481
1484
1487
1490
2921
2939
2948
2953
1361
1430
1444
1447
1457
1475
1480
1483
1486
1489
1492
2938
2945
1350
2922
2942
2920
2940
94
1358
2924
2944
2952
2949
99
FUPR686-09
PDCAP027-14
90
PDCAP028-14
FPSR296-10
FPSR293-10
99
FPSR294-10
FPSR295-10
FPSR297-10
FUPR685-09 P. corruscans
99
FUPR688-09|Zungaro jahu
0.020
S. scriptum
Upper Uruguay River
S. scriptum
Upper Paraná River
S. parahybae
