# Supplementary material for: Genetic Variation of the Endangered Neotropical Catfish Steindachneridion scriptum (Siluriformes: Pimelodidae)
Source: Front Genet. 2018 Feb 19;9:48. doi: 10.3389/fgene.2018.00048 (PMC5827538; doi:10.3389/fgene.2018.00048)

Supplementary Material

Genetic variation of the endangered Neotropical catfish *Steindachneridion scriptum* (Siluriformes: Pimelodidae)

Rômulo Veiga Paixão*, Josiane Ribolli, Evoy Zaniboni Filho

*** Correspondence:** Corresponding Author: romulo.veiga.paixao@gmail.com

1 Supplementary Information - SI

SI 2. Estimation of the probable groups of populations produced by the BAPS, using "clustering of individuals" option, between Steindachneridion scriptum from Upper Uruguay and Upper Paraná Basins, assigned to two clusters (K = 3).

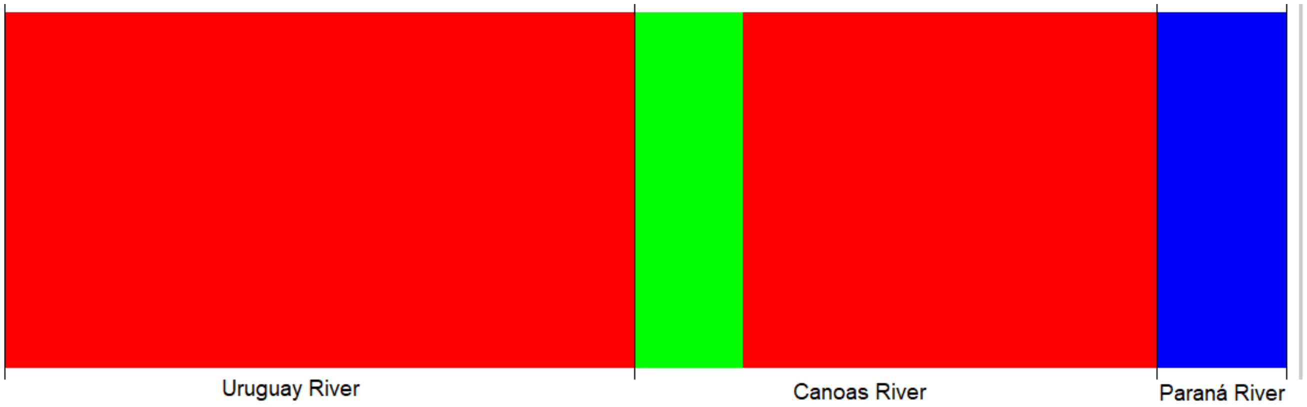

Supplement: Supplementary file 2 [file Image_2.pdf]
