# Supplementary material for: Genetic Variation of the Endangered Neotropical Catfish Steindachneridion scriptum (Siluriformes: Pimelodidae)
Source: Front Genet. 2018 Feb 19;9:48. doi: 10.3389/fgene.2018.00048 (PMC5827538; doi:10.3389/fgene.2018.00048)
Supplement: Supplementary file 3 [file Image_3.pdf]

Supplementary Material

Genetic variation of the endangered Neotropical catfish *Steindachneridion scriptum* (Siluriformes: Pimelodidae)

Rômulo Veiga Paixão*, Josiane Ribolli, Evoy Zaniboni Filho

*** Correspondence:** Corresponding Author: romulo.veiga.paixao@gmail.com

Supplementary Information - SI

SI 3. Pairwise Fst values plotted over distance reveal evident pattern of isolation by distance (r= 0.82, p= 0.0000) for Steindachneridion scriptum from Upper Uruguay and Upper Paraná basins, based on mtDNA control region haplotypes.

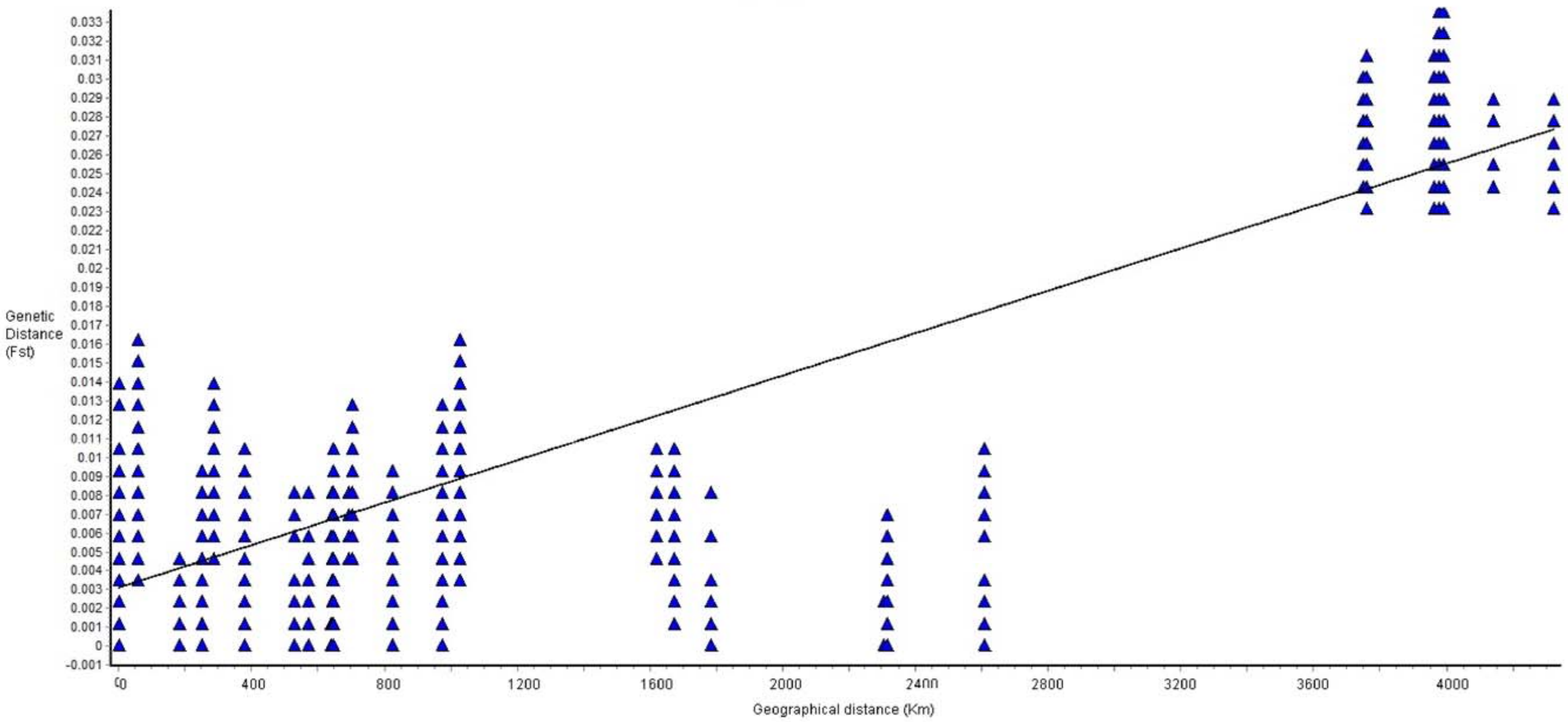

Genetic Distance (Fst)
0.033
0.032
0.031
0.03
0.029
0.028
0.027
0.026
0.025
0.024
0.023
0.022
0.021
0.02
0.019
0.018
0.017
0.016
0.015
0.014
0.013
0.012
0.011
0.01
0.009
0.008
0.007
0.006
0.005
0.004
0.003
0.002
0.001
0
-0.001
0
400
800
1200
1600
2000
2400
2800
3200
3600
4000
Geographical distance (Km)
