# Supplementary material for: Genetic Variation of the Endangered Neotropical Catfish Steindachneridion scriptum (Siluriformes: Pimelodidae)
Source: Front Genet. 2018 Feb 19;9:48. doi: 10.3389/fgene.2018.00048 (PMC5827538; doi:10.3389/fgene.2018.00048)

Supplementary Material

Genetic variation of the endangered Neotropical catfish *Steindachneridion scriptum* (Siluriformes: Pimelodidae)

Rômulo Veiga Paixão*, Josiane Ribolli, Evoy Zaniboni Filho

*** Correspondence:** Corresponding Author: romulo.veiga.paixao@gmail.com

Supplementary Information - SI

SI 4. Bayesian Skyline plot (BSP) showing the fluctuations over time in the effective size of the Upper Uruguay River populations. The black line represents the median and the blue area indicates 95% of the HPDs (highest posterior densities) of the estimates.

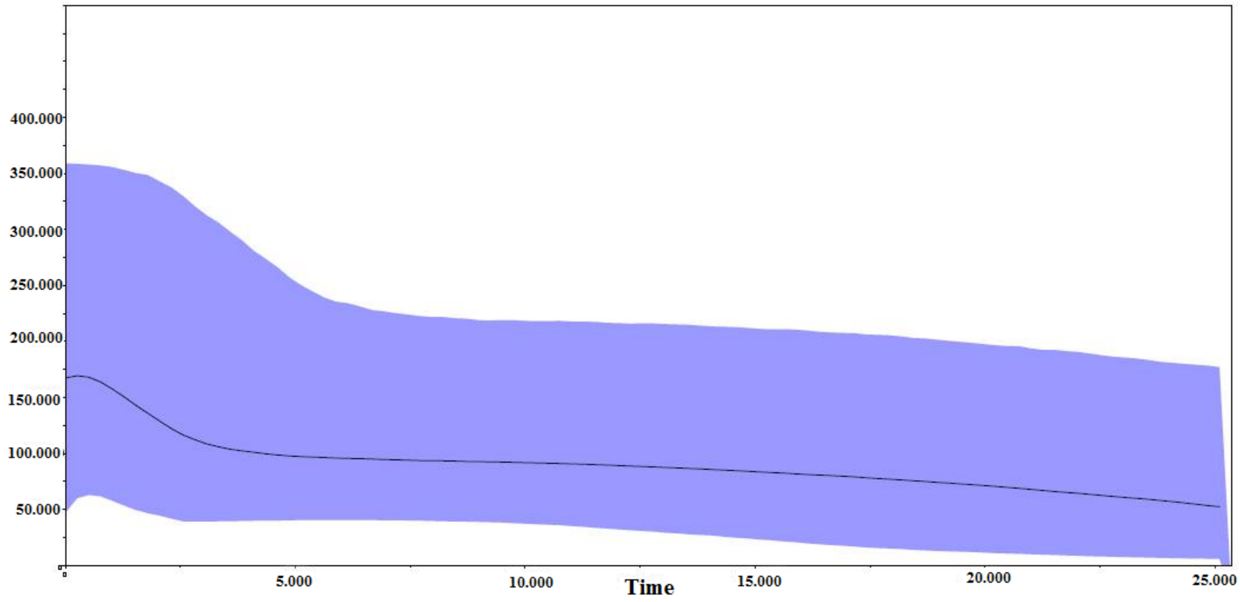

Supplement: Supplementary file 4 [file Image_4.PDF]
